# Octa-*n*-butyl-1κ^2^ *C*,2κ^2^ *C*,3κ^2^,4κ^2^ *C*-bis­(μ-2,3-dibromo­propionato)-1:2κ^2^ *O*:*O*′,3:4κ^2^ *O*:*O*′-bis­(2,3-dibromo­propionato)-1κ*O*,3κ*O*-di-μ_3_-oxido-1:2:4κ^3^ *O*:*O*:*O*,2:3:4κ^3^ *O*:*O*:*O*-tetra­tin(IV)

**DOI:** 10.1107/S1600536808037513

**Published:** 2008-11-20

**Authors:** Yip Foo Win, Siang Guan Teoh, Sie Tiong Ha, Reza Kia, Hoong-Kun Fun

**Affiliations:** a School of Chemical Sciences, Universiti Sains Malaysia, 11800 USM, Penang, Malaysia; b Universiti Tunku Abdul Rahman, Faculty of Engineering and Science, Jalan Genting Kelang, Setapak 53300, Kuala Lumpur, Malaysia; c X-ray Crystallography Unit, School of Physics, Universiti Sains Malaysia, 11800 USM, Penang, Malaysia

## Abstract

In the centrosymmetric tetra­nuclear title complex, [Sn_4_(C_4_H_9_)_8_(C_3_H_3_Br_2_O_2_)_4_O_2_], one of the two independent Sn atoms is five-coordinated by one O atom of the carboxyl­ate anion, two bridging O atoms and two *n*-butyl groups in a C_2_SnO_3_ distorted trigonal bipyramidal geometry. The other Sn atom also has a distorted trigonal bipyramidal geometry, being coordinated by two O atoms of two carboxyl­ate anions, one bridging O atom and two butyl groups. An inter­esting feature of the crystal structure is the short Sn⋯O [2.756 (4) Å] and O⋯O [2.608 (3) Å] inter­actions. The –BrCH_2_—CHBr– segments of the two carboxyl­ate anions are disordered over two positions [site occupancies of 0.60 (1)/0.40 (1) and 0.53 (2)/0.47 (2)]. Weak non-directional C—H⋯O inter­actions lead to the formation of infinte chains along the *a* axis; other weak inter­molecular C—H⋯π inter­actions are also present.

## Related literature

For hydrogen-bond motifs, see Bernstein *et al.* (1995 ▶). For bond-length data, see: Allen *et al.* (1987 ▶). For related distannoxanes, see: Gielen *et al.* (2000 ▶); Khan *et al.* (2000 ▶); Khoo & Hazell (1999 ▶); Li *et al.* (2006 ▶); Parvez *et al.* (2004 ▶); Ronconi *et al.* (2002 ▶); Tian *et al.* (2005 ▶); Win *et al.* (2008 ▶).
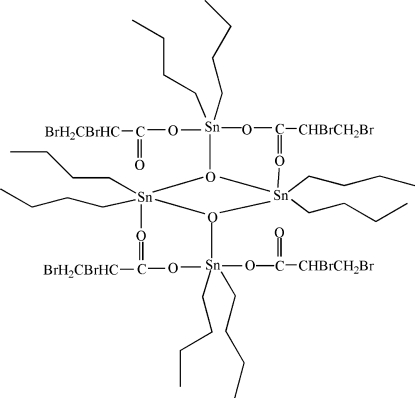


## Experimental

### 

#### Crystal data

[Sn_4_(C_4_H_9_)_8_(C_3_H_3_Br_2_O_2_)_4_O_2_]
*M* *_r_* = 1887.15
Monoclinic, 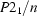

*a* = 11.7495 (4) Å
*b* = 20.6620 (8) Å
*c* = 12.9684 (5) Å
β = 91.462 (2)°
*V* = 3147.3 (2) Å^3^
*Z* = 2
Mo *K*α radiation
μ = 6.69 mm^−1^
*T* = 100.0 (1) K
0.51 × 0.32 × 0.25 mm

#### Data collection

Bruker SMART APEXII CCD area-detector diffractometer
Absorption correction: multi-scan (**SADABS**; Bruker, 2005 ▶) *T*_min_ = 0.088, *T*_max_ = 0.188
51856 measured reflections
12752 independent reflections
6843 reflections with *I* > 2σ(*I*)
*R*_int_ = 0.053

#### Refinement

*R*[*F* ^2^ > 2σ(*F* ^2^)] = 0.057
*wR*(*F* ^2^) = 0.144
*S* = 1.00
12752 reflections
365 parameters
12 restraints
H-atom parameters constrained
Δρ_max_ = 1.86 e Å^−3^
Δρ_min_ = −1.37 e Å^−3^

### 

Data collection: *APEX2* (Bruker, 2005 ▶); cell refinement: *APEX2*; data reduction: *SAINT* (Bruker, 2005 ▶); program(s) used to solve structure: *SIR2004* (Burla *et al.,* 2003 ▶); program(s) used to refine structure: *SHELXTL* (Sheldrick, 2008 ▶); molecular graphics: *SHELXTL*; software used to prepare material for publication: *SHELXTL* and *PLATON* (Spek, 2003 ▶).

## Supplementary Material

Crystal structure: contains datablocks global, I. DOI: 10.1107/S1600536808037513/ng2514sup1.cif

Structure factors: contains datablocks I. DOI: 10.1107/S1600536808037513/ng2514Isup2.hkl

Additional supplementary materials:  crystallographic information; 3D view; checkCIF report

## Figures and Tables

**Table 1:** Selected bond lengths (Å)

Sn1—O1	2.048 (3)
Sn1—C19	2.120 (5)
Sn1—C15	2.126 (5)
Sn1—O4	2.208 (3)
Sn1—O3	2.283 (4)
Sn2—O1	2.043 (3)
Sn2—C1	2.122 (6)
Sn2—C5	2.132 (6)
Sn2—O1^i^	2.149 (3)
Sn2—O2	2.300 (4)
Sn2—Sn2^i^	3.2840 (6)

Symmetry code: (i) 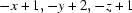.

**Table 2:** Hydrogen-bond geometry (Å, °) *Cg*1 and *Cg*2 are the centroids of the Sn2/O1/Sn2*A*/O1*A* and Sn1/O1/Sn2/O2/C9/O3 rings, respectively.

*D*—H⋯*A*	*D*—H	H⋯*A*	*D*⋯*A*	*D*—H⋯*A*
C6—H6*B*⋯O2	0.97	2.58	3.232 (9)	124
C14*A*—H14*A*⋯O3^ii^	0.97	2.56	3.434 (13)	149
C15—H15*A*⋯O5^ii^	0.97	2.53	3.220 (6)	128
C16—H16*A*⋯O3	0.97	2.45	3.134 (6)	127
C19—H19*A*⋯O5^ii^	0.97	2.57	3.287 (6)	130
C2—H2*A*⋯*Cg*1	0.97	2.95	3.415 (6)	111
C16—H16*A*⋯*Cg*2	0.97	2.68	3.250 (6)	118

Symmetry code: (ii) 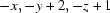.

**Fig. 1.**
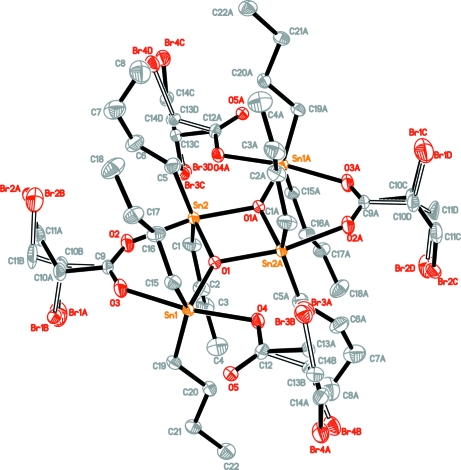
The molecular structure of the title compound with atom labels and 30% probability ellipsoids for non-H atoms. The H atoms were omitted for clarity. Solid bonds show the major disordered part.

**Fig. 2.**
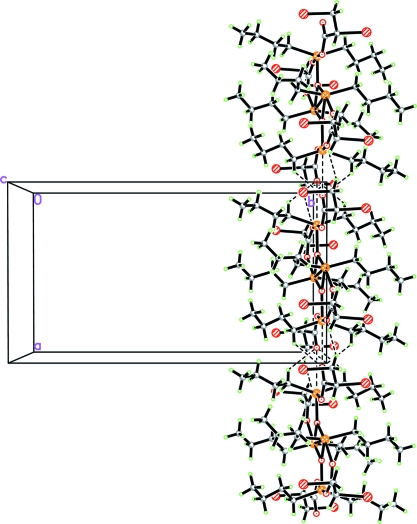
The crystal packing of the major component of (I), viewed down the c-axis, showing 1-D infinte chains along the a-axis. Intermolecular C—H···π interactions were shown as dashed lines.

## supplementary crystallographic information

### Comment

In recent years, different types of organotin(IV) complexes have been studied for their *in vitro* activity against a large array of tumor cell lines and have been found to be as effective as traditional heavy metal anticancer drugs, such as *cis*-platin and paraplatin (Gielen *et al.*, 2000; Khan *et al.*, 2000; Ronconi *et al.*, 2002; Tian *et al.*, 2005). In general, there are many well documented structures on complexes isolated from the 1:1 molar ratio reaction between diorganotin(IV) with the respective organic acids. Commonly, this dimeric structure is known as organodistannoxane dimer (Khoo & Hazell, 1999; Parvez *et al.*, 2004; Li *et al.*, 2006). The core geometry of the organodistannoxane dimer complexes consists of a centrosymmetric planar Sn_2_O_2_ group bonded to the *exo*- and endocyclic tin(IV) atom moiety *via* the bridging oxygen atoms so that the oxygen atoms are tri-coordinated. Recently, the crystal structure of the bis(2,4-dinitrobenzoato)tetrabutyldistannoxane(IV) dimer has been determined and consists of a centrosymmetric planar Sn_2_O_2_ group (Win *et al.*, 2008). In addition, all the four tin atoms (*exo*- and endocyclic) are five-coordinated and exist in distorted trigonal bypiramid geometry (Win *et al.*, 2008). In this study, the structure of the titled complex is similar to bis(2,4-dinitrobenzoato)tetrabutyldistannoxane(IV) dimer. The only exception is 2,3-dibromopropionic acid is utilized in the reaction to obtain the title complex.

The bond lengths (Allen *et al.*, 1987) and angles in the molecule (I, Fig. 1, Table 1) are within normal ranges. Intramolecular C—H···O hydrogen bonds generate *S(5)* ring motifs. In the title compound, one of the two independent Sn atoms is five-coordinated by the one oxygen atom of the carboxylate anoin, two oxo-bridged oxygen atoms and two *n*-butyl groups in a *trans*-C_2_SnO_3_ distorted trigonal-bipyramidal geometry. The other Sn atom has also a five-coordinated geometry which is coordinated by two oxygen atoms of the carboxylate anion, one oxo-bridged O atom and two butyl groups in a distorted trigonal-bipyramidal mode. The interesting feature of the crystal structure is the short Sn···O [2.756 (4)–3.271 (4) Å] and O···O [2.608 (3) Å], which are shorter than sum of the van der Waals radii of the relevant atoms (Spek, 2003). The –BrCH_2_—CHBr- segment of the carboxylate anion ligand is disordered over two positions with refined site-occupancies of 0.60 (1)/0.40 (1) and 0.53 (2)/0.47 (3), respectively. In the crystal structure, molecules are linked together through C—H···O hydrogen bonds, forming 1-D infinte chains along the *a* axis (Fig 2). The crystal structure is further stabilized by weak intermolecular C—H···π (Table 1) interactions.

### Experimental

The complex bis(2,3-dibromopropionato)tetrabutyldistannoxane(IV) dimer was obtained by heating under reflux a 1:1 molar mixture of dibutyltin(IV) oxide (0.50 g, 2 mmol) and 2,3-dibromopropionic acid (0.46 g, 2 mmol) in methanol (50 ml) for four hours. A clear colourless solution was isolated by filtration and kept in a bottle. After four days, colourless crystals (0.65 g, 69.4% yield) were collected. Melting point: 439.3 - 440.1 K. Analysis found for C_44_H_84_O_10_Br_8_Sn_4_: C, 28.36; H, 4.37; Sn, 24.97%. Calculated found for C_44_H_84_O_10_Br_8_Sn_4_: C, 28.00; H, 4.49; Sn, 25.16%. FTIR as KBr disc (cm^-1^): υ(C—H) saturated 2957, 2927, 2869; υ(COO)as 1654, 1614; υ(COO)s 1406, 1376; υ(Sn—O—Sn) 617; υ(Sn—O) 477.

### Refinement

All of the hydrogen atoms were positioned geometrically and refined using a riding model with C—H = 0.96–0.98 Å and *U*_iso_(H) = 1.2–1.5 *U*_eq_(C). A rotating group model was used for the methyl groups. The C–C bonds of the butyl groups were restrained to 1.500 (1) Å. The highest peak (1.86 e. Å^-3^) is located 0.71 Å from Sn1 and the deepest hole (-1.37 e. Å^-3^) is located 0.82 Å from Sn2. The C—C bonds in the butyl chains were restrained to 1.513 (2) Å.

### Crystal data

**Table:**

[Sn_4_(C_4_H_9_)_8_(C_3_H_3_Br_2_O_2_)_4_O_2_]	*F*_000_ = 1816
*M**_r_* = 1887.15	*D*_x_ = 1.991 Mg m^−^^3^
Monoclinic, *P*2_1_/*n*	Mo *K*α radiation λ = 0.71073 Å
Hall symbol: -P 2yn	Cell parameters from 9940 reflections
*a* = 11.7495 (4) Å	θ = 2.3–28.6º
*b* = 20.6620 (8) Å	µ = 6.69 mm^−^^1^
*c* = 12.9684 (5) Å	*T* = 100.0 (1) K
β = 91.462 (2)º	Block, colourless
*V* = 3147.3 (2) Å^3^	0.51 × 0.32 × 0.25 mm
*Z* = 2	

### Data collection

**Table:**

Bruker SMART APEXII CCD area-detector diffractometer	12752 independent reflections
Radiation source: fine-focus sealed tube	6843 reflections with *I* > 2σ(*I*)
Monochromator: graphite	*R*_int_ = 0.053
*T* = 100.0(1) K	θ_max_ = 34.0º
φ and ω scans	θ_min_ = 2.0º
Absorption correction: multi-scan(*SADABS*; Bruker, 2005)	*h* = −18→18
*T*_min_ = 0.088, *T*_max_ = 0.188	*k* = −24→32
51856 measured reflections	*l* = −16→20

### Refinement

**Table:**

Refinement on *F*^2^	Secondary atom site location: difference Fourier map
Least-squares matrix: full	Hydrogen site location: inferred from neighbouring sites
*R*[*F*^2^ > 2σ(*F*^2^)] = 0.057	H-atom parameters constrained
*wR*(*F*^2^) = 0.144	*w* = 1/[σ^2^(*F*_o_^2^) + (0.051*P*)^2^ + 7.7627*P*] where *P* = (*F*_o_^2^ + 2*F*_c_^2^)/3
*S* = 1.00	(Δ/σ)_max_ = 0.001
12752 reflections	Δρ_max_ = 1.86 e Å^−^^3^
365 parameters	Δρ_min_ = −1.37 e Å^−^^3^
12 restraints	Extinction correction: none
Primary atom site location: structure-invariant direct methods	

### Special details

**Table:**

Experimental. The low-temperature data was collected with the Oxford Cyrosystem Cobra low-temperature attachment.
Geometry. All e.s.d.'s (except the e.s.d. in the dihedral angle between two l.s. planes) are estimated using the full covariance matrix. The cell e.s.d.'s are taken into account individually in the estimation of e.s.d.'s in distances, angles and torsion angles; correlations between e.s.d.'s in cell parameters are only used when they are defined by crystal symmetry. An approximate (isotropic) treatment of cell e.s.d.'s is used for estimating e.s.d.'s involving l.s. planes.
Refinement. Refinement of *F*^2^ against ALL reflections. The weighted *R*-factor *wR* and goodness of fit *S* are based on *F*^2^, conventional *R*-factors *R* are based on *F*, with *F* set to zero for negative *F*^2^. The threshold expression of *F*^2^ > σ(*F*^2^) is used only for calculating *R*-factors(gt) *etc*. and is not relevant to the choice of reflections for refinement. *R*-factors based on *F*^2^ are statistically about twice as large as those based on *F*, and *R*- factors based on ALL data will be even larger.

### Fractional atomic coordinates and isotropic or equivalent isotropic displacement parameters (Å^2^)

**Table:**

	*x*	*y*	*z*	*U*_iso_*/*U*_eq_	Occ. (<1)
Sn1	0.21896 (3)	0.992516 (16)	0.45688 (2)	0.03422 (9)	
Sn2	0.52841 (3)	0.987445 (19)	0.37831 (2)	0.04044 (10)	
Br1A	0.2381 (4)	0.86283 (19)	0.1079 (3)	0.1169 (16)	0.604 (11)
Br2A	0.3252 (3)	1.07102 (19)	0.0148 (4)	0.0696 (9)	0.604 (11)
Br1B	0.2220 (3)	0.86703 (15)	0.1034 (2)	0.0426 (8)	0.396 (11)
Br2B	0.3090 (6)	1.0761 (3)	0.0346 (6)	0.0833 (18)	0.396 (11)
Br4A	0.0442 (9)	0.9327 (2)	0.8407 (5)	0.0672 (11)	0.53 (2)
Br3A	0.1343 (6)	1.1384 (3)	0.7509 (4)	0.0499 (7)	0.53 (2)
Br3B	0.1054 (7)	1.1495 (4)	0.7523 (5)	0.0516 (8)	0.47 (2)
Br4B	0.0709 (4)	0.9362 (3)	0.8620 (6)	0.0616 (8)	0.47 (2)
O1	0.3928 (3)	0.99367 (16)	0.4734 (2)	0.0348 (7)	
O2	0.4051 (4)	0.9684 (3)	0.2411 (3)	0.0657 (9)	
O3	0.2282 (4)	0.9870 (2)	0.2815 (3)	0.0657 (9)	
O4	0.2381 (3)	1.00102 (18)	0.6261 (3)	0.0423 (8)	
O5	0.0507 (3)	1.00432 (16)	0.6120 (3)	0.0388 (7)	
C1	0.5744 (5)	0.8886 (3)	0.3650 (5)	0.0624 (17)	
H1A	0.5767	0.8773	0.2925	0.075*	
H1B	0.6503	0.8826	0.3946	0.075*	
C2	0.4935 (4)	0.8430 (2)	0.4179 (5)	0.0586 (15)	
H2A	0.4840	0.8579	0.4881	0.070*	
H2B	0.4198	0.8456	0.3827	0.070*	
C3	0.5307 (5)	0.7729 (2)	0.4209 (5)	0.0672 (18)	
H3A	0.5338	0.7569	0.3508	0.081*	
H3B	0.6072	0.7707	0.4508	0.081*	
C4	0.4541 (6)	0.7291 (3)	0.4817 (6)	0.081 (2)	
H4A	0.4778	0.6850	0.4737	0.121*	
H4B	0.3769	0.7338	0.4566	0.121*	
H4C	0.4588	0.7408	0.5533	0.121*	
C5	0.5498 (5)	1.0807 (3)	0.3110 (4)	0.0559 (15)	
H5A	0.4780	1.1036	0.3142	0.067*	
H5B	0.6051	1.1045	0.3529	0.067*	
C6	0.5880 (5)	1.0815 (3)	0.2005 (4)	0.073 (2)	
H6A	0.6663	1.0667	0.1992	0.088*	
H6B	0.5420	1.0509	0.1611	0.088*	
C7	0.5806 (6)	1.1466 (3)	0.1475 (6)	0.086 (2)	
H7A	0.5911	1.1407	0.0741	0.103*	
H7B	0.5052	1.1646	0.1565	0.103*	
C8	0.6685 (7)	1.1941 (4)	0.1888 (7)	0.099 (3)	
H8A	0.6716	1.2309	0.1438	0.148*	
H8B	0.7417	1.1735	0.1924	0.148*	
H8C	0.6480	1.2080	0.2565	0.148*	
C9	0.3011 (5)	0.9730 (3)	0.2232 (4)	0.0560 (16)	
C10A	0.2518 (10)	0.9633 (8)	0.1134 (10)	0.052 (2)	0.604 (11)
H10A	0.1782	0.9850	0.1026	0.063*	0.604 (11)
C11A	0.3317 (9)	0.9767 (7)	0.0293 (7)	0.073 (4)	0.604 (11)
H11A	0.3072	0.9554	−0.0341	0.087*	0.604 (11)
H11B	0.4081	0.9624	0.0480	0.087*	0.604 (11)
C10B	0.2938 (16)	0.9483 (12)	0.1031 (16)	0.052 (2)	0.396 (11)
H10B	0.3697	0.9454	0.0738	0.063*	0.396 (11)
C11B	0.2249 (14)	1.0033 (14)	0.0539 (12)	0.108 (11)	0.396 (11)
H11C	0.1616	1.0136	0.0975	0.129*	0.396 (11)
H11D	0.1938	0.9889	−0.0122	0.129*	0.396 (11)
C12	0.1386 (4)	1.0095 (3)	0.6628 (4)	0.0411 (11)	
C13A	0.1425 (11)	1.0446 (8)	0.7698 (11)	0.041 (3)	0.53 (2)
H13A	0.2120	1.0328	0.8088	0.049*	0.53 (2)
C13B	0.1286 (12)	1.0153 (9)	0.7786 (11)	0.039 (3)	0.47 (2)
H13B	0.2038	1.0272	0.8071	0.046*	0.47 (2)
C14A	0.0398 (10)	1.0263 (8)	0.8286 (9)	0.050 (4)	0.53 (2)
H14A	−0.0291	1.0400	0.7920	0.060*	0.53 (2)
H14B	0.0420	1.0462	0.8963	0.060*	0.53 (2)
C14B	0.0472 (10)	1.0681 (8)	0.8062 (10)	0.049 (4)	0.47 (2)
H14C	0.0409	1.0708	0.8805	0.059*	0.47 (2)
H14D	−0.0277	1.0592	0.7763	0.059*	0.47 (2)
C15	0.1635 (4)	1.0893 (2)	0.4323 (4)	0.0440 (11)	
H15A	0.0881	1.0885	0.4000	0.053*	
H15B	0.1574	1.1106	0.4987	0.053*	
C16	0.2414 (4)	1.1288 (2)	0.3657 (4)	0.0505 (13)	
H16A	0.2620	1.1029	0.3067	0.061*	
H16B	0.3108	1.1381	0.4051	0.061*	
C17	0.1913 (5)	1.1921 (2)	0.3270 (5)	0.0576 (15)	
H17A	0.1180	1.1837	0.2934	0.069*	
H17B	0.1788	1.2204	0.3853	0.069*	
C18	0.2681 (7)	1.2257 (3)	0.2519 (5)	0.080 (2)	
H18A	0.2330	1.2652	0.2289	0.121*	
H18B	0.2800	1.1979	0.1938	0.121*	
H18C	0.3399	1.2351	0.2855	0.121*	
C19	0.1594 (4)	0.8959 (2)	0.4437 (3)	0.0414 (11)	
H19A	0.0807	0.8978	0.4195	0.050*	
H19B	0.2021	0.8752	0.3898	0.050*	
C20	0.1642 (4)	0.85156 (19)	0.5366 (3)	0.0422 (11)	
H20A	0.1293	0.8731	0.5943	0.051*	
H20B	0.2431	0.8430	0.5557	0.051*	
C21	0.1037 (4)	0.78800 (19)	0.5159 (4)	0.0465 (12)	
H21A	0.0244	0.7968	0.4989	0.056*	
H21B	0.1369	0.7676	0.4564	0.056*	
C22	0.1099 (5)	0.7412 (3)	0.6057 (4)	0.0600 (15)	
H22A	0.0682	0.7027	0.5881	0.090*	
H22B	0.0775	0.7610	0.6651	0.090*	
H22C	0.1880	0.7303	0.6207	0.090*	

### Atomic displacement parameters (Å^2^)

**Table:**

	*U*^11^	*U*^22^	*U*^33^	*U*^12^	*U*^13^	*U*^23^
Sn1	0.03673 (15)	0.03153 (17)	0.03398 (16)	−0.00327 (12)	−0.00747 (11)	0.00247 (13)
Sn2	0.03953 (16)	0.0544 (2)	0.02729 (15)	−0.01181 (14)	−0.00053 (12)	−0.00482 (14)
Br1A	0.196 (3)	0.0626 (17)	0.0895 (19)	−0.0263 (17)	−0.049 (2)	0.0114 (13)
Br2A	0.0805 (12)	0.0672 (18)	0.0593 (14)	−0.0346 (12)	−0.0307 (10)	0.0210 (12)
Br1B	0.0681 (15)	0.0231 (11)	0.0366 (12)	−0.0071 (8)	0.0018 (9)	−0.0047 (8)
Br2B	0.142 (4)	0.0406 (17)	0.066 (3)	0.0102 (19)	−0.026 (2)	−0.0053 (16)
Br4A	0.095 (3)	0.0468 (9)	0.0590 (16)	−0.0083 (14)	−0.0212 (14)	0.0068 (9)
Br3A	0.0560 (19)	0.0349 (15)	0.0587 (9)	−0.0054 (10)	−0.0009 (13)	−0.0095 (9)
Br3B	0.059 (2)	0.0401 (17)	0.0549 (10)	−0.0037 (13)	−0.0041 (14)	−0.0100 (11)
Br4B	0.0625 (14)	0.0550 (13)	0.066 (2)	−0.0029 (9)	−0.0291 (10)	0.0088 (13)
O1	0.0345 (14)	0.0381 (18)	0.0316 (15)	−0.0049 (12)	−0.0033 (12)	−0.0015 (13)
O2	0.0628 (18)	0.097 (3)	0.0367 (15)	−0.0153 (17)	−0.0076 (13)	−0.0095 (15)
O3	0.0628 (18)	0.097 (3)	0.0367 (15)	−0.0153 (17)	−0.0076 (13)	−0.0095 (15)
O4	0.0366 (16)	0.054 (2)	0.0363 (18)	0.0012 (14)	−0.0051 (13)	−0.0023 (15)
O5	0.0354 (15)	0.041 (2)	0.0397 (18)	−0.0026 (13)	−0.0013 (13)	−0.0007 (14)
C1	0.060 (3)	0.057 (4)	0.071 (4)	−0.017 (3)	0.024 (3)	−0.027 (3)
C2	0.051 (3)	0.056 (4)	0.068 (4)	−0.003 (3)	0.004 (3)	−0.011 (3)
C3	0.067 (4)	0.057 (4)	0.079 (5)	−0.003 (3)	0.010 (3)	−0.026 (3)
C4	0.082 (5)	0.056 (4)	0.104 (6)	0.012 (4)	0.012 (4)	0.006 (4)
C5	0.048 (3)	0.065 (4)	0.054 (3)	−0.001 (3)	−0.006 (2)	0.018 (3)
C6	0.055 (3)	0.095 (6)	0.069 (4)	−0.002 (3)	0.005 (3)	0.033 (4)
C7	0.069 (4)	0.088 (6)	0.100 (6)	0.005 (4)	−0.001 (4)	0.032 (5)
C8	0.134 (8)	0.065 (5)	0.097 (6)	0.002 (5)	−0.019 (6)	−0.004 (5)
C9	0.074 (4)	0.065 (4)	0.028 (2)	−0.033 (3)	−0.011 (2)	0.004 (2)
C10A	0.046 (7)	0.080 (8)	0.030 (4)	0.001 (6)	−0.001 (5)	−0.012 (4)
C11A	0.053 (6)	0.130 (11)	0.035 (5)	0.002 (6)	−0.007 (4)	−0.021 (6)
C10B	0.046 (7)	0.080 (8)	0.030 (4)	0.001 (6)	−0.001 (5)	−0.012 (4)
C11B	0.050 (9)	0.24 (3)	0.029 (7)	0.012 (13)	−0.015 (6)	0.022 (12)
C12	0.042 (2)	0.047 (3)	0.034 (2)	0.005 (2)	0.0031 (19)	0.002 (2)
C13A	0.041 (6)	0.046 (8)	0.034 (6)	−0.005 (5)	−0.006 (4)	−0.001 (6)
C13B	0.038 (5)	0.052 (10)	0.027 (5)	−0.008 (6)	0.009 (4)	0.001 (7)
C14A	0.061 (6)	0.048 (9)	0.040 (6)	−0.001 (5)	0.005 (5)	0.003 (5)
C14B	0.044 (6)	0.054 (10)	0.051 (7)	−0.009 (5)	0.004 (5)	0.001 (6)
C15	0.043 (3)	0.034 (3)	0.055 (3)	0.0002 (19)	−0.005 (2)	0.007 (2)
C16	0.054 (3)	0.038 (3)	0.060 (3)	−0.002 (2)	0.005 (3)	0.006 (3)
C17	0.071 (4)	0.036 (3)	0.066 (4)	0.001 (3)	0.010 (3)	0.007 (3)
C18	0.126 (6)	0.044 (4)	0.072 (5)	0.004 (4)	0.028 (4)	0.012 (3)
C19	0.039 (2)	0.035 (3)	0.050 (3)	−0.0035 (18)	−0.007 (2)	0.000 (2)
C20	0.045 (2)	0.038 (3)	0.044 (3)	0.001 (2)	−0.007 (2)	−0.001 (2)
C21	0.051 (3)	0.032 (3)	0.056 (3)	−0.004 (2)	0.000 (2)	−0.004 (2)
C22	0.067 (4)	0.040 (3)	0.073 (4)	−0.003 (3)	−0.002 (3)	0.009 (3)

### Geometric parameters (Å, °)

**Table:**

Sn1—O1	2.048 (3)	C8—H8B	0.9600
Sn1—C19	2.120 (5)	C8—H8C	0.9600
Sn1—C15	2.126 (5)	C9—C10A	1.537 (13)
Sn1—O4	2.208 (3)	C9—C10B	1.64 (2)
Sn1—O3	2.283 (4)	C10A—C11A	1.483 (16)
Sn2—O1	2.043 (3)	C10A—H10A	0.9800
Sn2—C1	2.122 (6)	C11A—H11A	0.9700
Sn2—C5	2.132 (6)	C11A—H11B	0.9700
Sn2—O1^i^	2.149 (3)	C10B—C11B	1.53 (3)
Sn2—O2	2.300 (4)	C10B—H10B	0.9800
Sn2—Sn2^i^	3.2840 (6)	C11B—H11C	0.9700
Br1A—C10A	2.084 (16)	C11B—H11D	0.9700
Br2A—C11A	1.960 (15)	C12—C13B	1.513 (15)
Br1B—C10B	1.88 (3)	C12—C13A	1.565 (15)
Br2B—C11B	1.82 (3)	C13A—C14A	1.49 (2)
Br4A—C14A	1.940 (17)	C13A—H13A	0.9800
Br3A—C13A	1.956 (19)	C13B—C14B	1.50 (2)
Br3B—C14B	1.952 (17)	C13B—H13B	0.9800
Br4B—C13B	2.083 (17)	C14A—H14A	0.9700
O1—Sn2^i^	2.149 (3)	C14A—H14B	0.9700
O2—C9	1.241 (7)	C14B—H14C	0.9700
O3—C9	1.192 (7)	C14B—H14D	0.9700
O4—C12	1.285 (6)	C15—C16	1.513 (2)
O5—C12	1.216 (6)	C15—H15A	0.9700
C1—C2	1.513 (2)	C15—H15B	0.9700
C1—H1A	0.9700	C16—C17	1.513 (2)
C1—H1B	0.9700	C16—H16A	0.9700
C2—C3	1.513 (2)	C16—H16B	0.9700
C2—H2A	0.9700	C17—C18	1.513 (2)
C2—H2B	0.9700	C17—H17A	0.9700
C3—C4	1.513 (2)	C17—H17B	0.9700
C3—H3A	0.9700	C18—H18A	0.9600
C3—H3B	0.9700	C18—H18B	0.9600
C4—H4A	0.9600	C18—H18C	0.9600
C4—H4B	0.9600	C19—C20	1.513 (2)
C4—H4C	0.9600	C19—H19A	0.9700
C5—C6	1.513 (2)	C19—H19B	0.9700
C5—H5A	0.9700	C20—C21	1.514 (2)
C5—H5B	0.9700	C20—H20A	0.9700
C6—C7	1.513 (2)	C20—H20B	0.9700
C6—H6A	0.9700	C21—C22	1.513 (2)
C6—H6B	0.9700	C21—H21A	0.9700
C7—C8	1.512 (2)	C21—H21B	0.9700
C7—H7A	0.9700	C22—H22A	0.9600
C7—H7B	0.9700	C22—H22B	0.9600
C8—H8A	0.9600	C22—H22C	0.9600
			
O1—Sn1—C19	110.21 (16)	C10A—C11A—H11B	111.1
O1—Sn1—C15	107.81 (15)	Br2A—C11A—H11B	111.1
C19—Sn1—C15	140.76 (18)	H11A—C11A—H11B	109.0
O1—Sn1—O4	79.58 (12)	C11B—C10B—C9	100.3 (14)
C19—Sn1—O4	100.37 (15)	C11B—C10B—Br1B	115.7 (15)
C15—Sn1—O4	95.58 (17)	C9—C10B—Br1B	106.8 (13)
O1—Sn1—O3	91.83 (14)	C11B—C10B—H10B	111.1
C19—Sn1—O3	84.07 (17)	C9—C10B—H10B	111.1
C15—Sn1—O3	85.41 (19)	Br1B—C10B—H10B	111.1
O4—Sn1—O3	171.25 (14)	C10B—C11B—Br2B	112.9 (13)
O1—Sn2—C1	108.28 (15)	C10B—C11B—H11C	109.0
O1—Sn2—C5	106.96 (17)	Br2B—C11B—H11C	109.0
C1—Sn2—C5	143.5 (2)	C10B—C11B—H11D	109.0
O1—Sn2—O1^i^	76.90 (13)	Br2B—C11B—H11D	109.0
C1—Sn2—O1^i^	98.2 (2)	H11C—C11B—H11D	107.8
C5—Sn2—O1^i^	98.61 (17)	O5—C12—O4	123.7 (5)
O1—Sn2—O2	89.54 (14)	O5—C12—C13B	117.3 (7)
C1—Sn2—O2	85.9 (2)	O4—C12—C13B	118.2 (7)
C5—Sn2—O2	85.2 (2)	O5—C12—C13A	121.8 (6)
O1^i^—Sn2—O2	166.43 (14)	O4—C12—C13A	112.7 (6)
O1—Sn2—Sn2^i^	39.59 (8)	C13B—C12—C13A	23.8 (5)
C1—Sn2—Sn2^i^	106.78 (16)	C14A—C13A—C12	109.3 (11)
C5—Sn2—Sn2^i^	106.23 (15)	C14A—C13A—Br3A	106.1 (12)
O1^i^—Sn2—Sn2^i^	37.30 (8)	C12—C13A—Br3A	110.3 (9)
O2—Sn2—Sn2^i^	129.13 (11)	C14A—C13A—H13A	110.4
Sn2—O1—Sn1	136.64 (16)	C12—C13A—H13A	110.4
Sn2—O1—Sn2^i^	103.10 (12)	Br3A—C13A—H13A	110.4
Sn1—O1—Sn2^i^	120.08 (15)	C14B—C13B—C12	111.2 (13)
C9—O2—Sn2	136.8 (4)	C14B—C13B—Br4B	103.2 (10)
C9—O3—Sn1	134.2 (4)	C12—C13B—Br4B	119.1 (12)
C12—O4—Sn1	108.0 (3)	C14B—C13B—H13B	107.6
C2—C1—Sn2	113.4 (4)	C12—C13B—H13B	107.6
C2—C1—H1A	108.9	Br4B—C13B—H13B	107.6
Sn2—C1—H1A	108.9	C13A—C14A—Br4A	105.9 (11)
C2—C1—H1B	108.9	C13A—C14A—H14A	110.6
Sn2—C1—H1B	108.9	Br4A—C14A—H14A	110.6
H1A—C1—H1B	107.7	C13A—C14A—H14B	110.6
C3—C2—C1	115.0 (5)	Br4A—C14A—H14B	110.6
C3—C2—H2A	108.5	H14A—C14A—H14B	108.7
C1—C2—H2A	108.5	C13B—C14B—Br3B	108.0 (12)
C3—C2—H2B	108.5	C13B—C14B—H14C	110.1
C1—C2—H2B	108.5	Br3B—C14B—H14C	110.1
H2A—C2—H2B	107.5	C13B—C14B—H14D	110.1
C4—C3—C2	114.3 (5)	Br3B—C14B—H14D	110.1
C4—C3—H3A	108.7	H14C—C14B—H14D	108.4
C2—C3—H3A	108.7	C16—C15—Sn1	113.9 (3)
C4—C3—H3B	108.7	C16—C15—H15A	108.8
C2—C3—H3B	108.7	Sn1—C15—H15A	108.8
H3A—C3—H3B	107.6	C16—C15—H15B	108.8
C3—C4—H4A	109.5	Sn1—C15—H15B	108.8
C3—C4—H4B	109.5	H15A—C15—H15B	107.7
H4A—C4—H4B	109.5	C15—C16—C17	114.8 (4)
C3—C4—H4C	109.5	C15—C16—H16A	108.6
H4A—C4—H4C	109.5	C17—C16—H16A	108.6
H4B—C4—H4C	109.5	C15—C16—H16B	108.6
C6—C5—Sn2	116.1 (4)	C17—C16—H16B	108.6
C6—C5—H5A	108.3	H16A—C16—H16B	107.6
Sn2—C5—H5A	108.3	C18—C17—C16	112.1 (5)
C6—C5—H5B	108.3	C18—C17—H17A	109.2
Sn2—C5—H5B	108.3	C16—C17—H17A	109.2
H5A—C5—H5B	107.4	C18—C17—H17B	109.2
C7—C6—C5	115.2 (6)	C16—C17—H17B	109.2
C7—C6—H6A	108.5	H17A—C17—H17B	107.9
C5—C6—H6A	108.5	C17—C18—H18A	109.5
C7—C6—H6B	108.5	C17—C18—H18B	109.5
C5—C6—H6B	108.5	H18A—C18—H18B	109.5
H6A—C6—H6B	107.5	C17—C18—H18C	109.5
C8—C7—C6	112.6 (6)	H18A—C18—H18C	109.5
C8—C7—H7A	109.1	H18B—C18—H18C	109.5
C6—C7—H7A	109.1	C20—C19—Sn1	120.1 (3)
C8—C7—H7B	109.1	C20—C19—H19A	107.3
C6—C7—H7B	109.1	Sn1—C19—H19A	107.3
H7A—C7—H7B	107.8	C20—C19—H19B	107.3
C7—C8—H8A	109.5	Sn1—C19—H19B	107.3
C7—C8—H8B	109.5	H19A—C19—H19B	106.9
H8A—C8—H8B	109.5	C19—C20—C21	112.2 (4)
C7—C8—H8C	109.5	C19—C20—H20A	109.2
H8A—C8—H8C	109.5	C21—C20—H20A	109.2
H8B—C8—H8C	109.5	C19—C20—H20B	109.2
O3—C9—O2	128.3 (5)	C21—C20—H20B	109.2
O3—C9—C10A	111.0 (7)	H20A—C20—H20B	107.9
O2—C9—C10A	120.6 (7)	C22—C21—C20	113.9 (4)
O3—C9—C10B	131.0 (8)	C22—C21—H21A	108.8
O2—C9—C10B	100.4 (8)	C20—C21—H21A	108.8
C10A—C9—C10B	21.5 (6)	C22—C21—H21B	108.8
C11A—C10A—C9	115.2 (9)	C20—C21—H21B	108.8
C11A—C10A—Br1A	102.1 (9)	H21A—C21—H21B	107.7
C9—C10A—Br1A	100.9 (8)	C21—C22—H22A	109.5
C11A—C10A—H10A	112.5	C21—C22—H22B	109.5
C9—C10A—H10A	112.5	H22A—C22—H22B	109.5
Br1A—C10A—H10A	112.5	C21—C22—H22C	109.5
C10A—C11A—Br2A	103.4 (9)	H22A—C22—H22C	109.5
C10A—C11A—H11A	111.1	H22B—C22—H22C	109.5
Br2A—C11A—H11A	111.1		
			
C1—Sn2—O1—Sn1	−90.7 (3)	Sn2—O2—C9—C10B	180.0 (10)
C5—Sn2—O1—Sn1	79.7 (3)	O3—C9—C10A—C11A	152.2 (10)
O1^i^—Sn2—O1—Sn1	174.9 (3)	O2—C9—C10A—C11A	−25.3 (15)
O2—Sn2—O1—Sn1	−5.1 (3)	C10B—C9—C10A—C11A	−47 (3)
Sn2^i^—Sn2—O1—Sn1	174.9 (3)	O3—C9—C10A—Br1A	−98.7 (7)
C1—Sn2—O1—Sn2^i^	94.4 (2)	O2—C9—C10A—Br1A	83.8 (8)
C5—Sn2—O1—Sn2^i^	−95.15 (19)	C10B—C9—C10A—Br1A	62 (4)
O1^i^—Sn2—O1—Sn2^i^	0.0	C9—C10A—C11A—Br2A	−81.7 (11)
O2—Sn2—O1—Sn2^i^	−179.98 (17)	Br1A—C10A—C11A—Br2A	170.0 (5)
C19—Sn1—O1—Sn2	81.2 (3)	O3—C9—C10B—C11B	56 (2)
C15—Sn1—O1—Sn2	−88.9 (3)	O2—C9—C10B—C11B	−129.2 (13)
O4—Sn1—O1—Sn2	178.5 (3)	C10A—C9—C10B—C11B	32 (3)
O3—Sn1—O1—Sn2	−3.1 (3)	O3—C9—C10B—Br1B	−65.0 (16)
C19—Sn1—O1—Sn2^i^	−104.5 (2)	O2—C9—C10B—Br1B	109.7 (9)
C15—Sn1—O1—Sn2^i^	85.4 (2)	C10A—C9—C10B—Br1B	−89 (4)
O4—Sn1—O1—Sn2^i^	−7.20 (16)	C9—C10B—C11B—Br2B	76.4 (15)
O3—Sn1—O1—Sn2^i^	171.1 (2)	Br1B—C10B—C11B—Br2B	−169.1 (10)
O1—Sn2—O2—C9	13.7 (6)	Sn1—O4—C12—O5	9.2 (6)
C1—Sn2—O2—C9	122.0 (7)	Sn1—O4—C12—C13B	178.7 (8)
C5—Sn2—O2—C9	−93.4 (7)	Sn1—O4—C12—C13A	−155.6 (7)
O1^i^—Sn2—O2—C9	13.6 (12)	O5—C12—C13A—C14A	41.0 (15)
Sn2^i^—Sn2—O2—C9	13.7 (7)	O4—C12—C13A—C14A	−153.9 (10)
O1—Sn1—O3—C9	17.4 (6)	C13B—C12—C13A—C14A	−45 (2)
C19—Sn1—O3—C9	−92.8 (6)	O5—C12—C13A—Br3A	−75.3 (9)
C15—Sn1—O3—C9	125.1 (6)	O4—C12—C13A—Br3A	89.8 (8)
O4—Sn1—O3—C9	28.2 (15)	C13B—C12—C13A—Br3A	−161 (3)
O1—Sn1—O4—C12	173.4 (3)	O5—C12—C13B—C14B	−52.0 (15)
C19—Sn1—O4—C12	−77.8 (3)	O4—C12—C13B—C14B	137.8 (10)
C15—Sn1—O4—C12	66.2 (3)	C13A—C12—C13B—C14B	55 (2)
O3—Sn1—O4—C12	162.3 (10)	O5—C12—C13B—Br4B	67.7 (11)
O1—Sn2—C1—C2	5.6 (5)	O4—C12—C13B—Br4B	−102.5 (9)
C5—Sn2—C1—C2	−158.9 (4)	C13A—C12—C13B—Br4B	175 (3)
O1^i^—Sn2—C1—C2	84.4 (5)	C12—C13A—C14A—Br4A	58.1 (13)
O2—Sn2—C1—C2	−82.6 (5)	Br3A—C13A—C14A—Br4A	177.1 (6)
Sn2^i^—Sn2—C1—C2	47.2 (5)	C12—C13B—C14B—Br3B	−60.6 (13)
Sn2—C1—C2—C3	−173.2 (5)	Br4B—C13B—C14B—Br3B	170.5 (7)
C1—C2—C3—C4	175.2 (6)	O1—Sn1—C15—C16	37.9 (4)
O1—Sn2—C5—C6	−144.3 (4)	C19—Sn1—C15—C16	−127.4 (4)
C1—Sn2—C5—C6	20.3 (7)	O4—Sn1—C15—C16	118.7 (4)
O1^i^—Sn2—C5—C6	136.9 (4)	O3—Sn1—C15—C16	−52.6 (4)
O2—Sn2—C5—C6	−56.2 (4)	Sn1—C15—C16—C17	166.7 (4)
Sn2^i^—Sn2—C5—C6	174.4 (4)	C15—C16—C17—C18	−173.8 (6)
Sn2—C5—C6—C7	168.6 (4)	O1—Sn1—C19—C20	70.9 (4)
C5—C6—C7—C8	70.7 (9)	C15—Sn1—C19—C20	−124.0 (4)
Sn1—O3—C9—O2	−15.3 (11)	O4—Sn1—C19—C20	−11.7 (4)
Sn1—O3—C9—C10A	167.4 (7)	O3—Sn1—C19—C20	160.7 (4)
Sn1—O3—C9—C10B	158.1 (13)	Sn1—C19—C20—C21	172.3 (3)
Sn2—O2—C9—O3	−5.1 (12)	C19—C20—C21—C22	178.0 (5)
Sn2—O2—C9—C10A	172.0 (8)		

Symmetry codes: (i) −*x*+1, −*y*+2, −*z*+1.

### Hydrogen-bond geometry (Å, °)

**Table:**

*D*—H···*A*	*D*—H	H···*A*	*D*···*A*	*D*—H···*A*
C6—H6B···O2	0.97	2.58	3.232 (9)	124
C14A—H14A···O3^ii^	0.97	2.56	3.434 (13)	149
C15—H15A···O5^ii^	0.97	2.53	3.220 (6)	128
C16—H16A···O3	0.97	2.45	3.134 (6)	127
C19—H19A···O5^ii^	0.97	2.57	3.287 (6)	130
C2—H2A···Cg1	0.97	2.95	3.415 (6)	111
C16—H16A···Cg2	0.97	2.68	3.250 (6)	118

Symmetry codes: (ii) −*x*, −*y*+2, −*z*+1.
